# Do Absorbable Sutures Work for Rectus Diastasis Repair in Abdominoplasty Patients?

**DOI:** 10.1093/asjof/ojae040

**Published:** 2024-05-23

**Authors:** Brandon T Jackson, Simon Moradian, Jonathan T Bricker, Kareem M Termanini, Sarah Ferenz, Jennifer Bai, John Y Kim

## Abstract

**Background:**

The standard treatment for rectus diastasis is rectus sheath plication during abdominoplasty. Lasting correction of diastasis is essential, but there is currently a debate as to whether absorbable or nonabsorbable rectus plication achieves a lower rate of recurrence.

**Objectives:**

The goal of this study is to assess long-term patient outcomes and the recurrence of rectus diastasis after plication with long-lasting absorbable sutures.

**Methods:**

A retrospective study of abdominoplasties performed by the senior author between 2018 and 2022 was performed. Only female patients with >6 months of follow-up were included. Plication of the rectus muscles was performed with a combination of interrupted, buried, figure of eight #0 polydioxanone suture and running #0 Maxon (Covidien, Mansfield, MA). Outcomes were assessed by physical examination at postoperative visits. A retrospective chart review was used to obtain demographic and perioperative information.

**Results:**

Seventy-one patients underwent abdominoplasty with an average follow-up of 21.1 months. The average age was 43 years, and the average BMI was 27 kg/m^2^. Correction of rectus diastasis was performed using absorbable sutures in all patients with no recurrence of diastasis in any patient (0% diastasis recurrence rate). Complications included delayed wound healing (11%), seroma (8.5%), hematoma (2.8%), and deep vein thrombosis/pulmonary embolism (2.8%). No patients needed reoperation.

**Conclusions:**

Abdominal wall plication using a double-layered, long-lasting absorbable suture closure is a safe, reliable, and effective method to address rectus diastasis during abdominoplasty. Our technique achieved no recurrence of diastasis in any patient and a low complication profile.

**Level of Evidence: 3:**

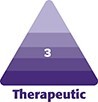

Rectus diastasis is a relatively common condition encountered by plastic surgeons in which there is a widening of the linea alba along its length due to midline separation of the rectus abdominis muscles by an abnormal distance. This defect is generally caused by an increase in intra-abdominal pressure, which applies force to the linea alba and causes stretching. This is often observed following pregnancy, although other causes include obesity, prior abdominal operations, and congenital abnormalities.^1-4^ Clinical examination is generally sufficient for diagnosis, but it may be supplemented with ultrasound, computed tomography (CT), or MRI. Because rectus diastasis can result in a protruding abdomen, causing both functional and cosmetic complaints, surgical correction is often the appropriate method of treatment.^1,5-7^

Rectus sheath plication during abdominoplasty is the standard treatment for diastasis of the rectus muscles to improve abdominal contour. Essential to this repair is a lasting correction of this diastasis using sutures to reapproximate the rectus muscles along the midline. This can be achieved through several surgical techniques, including single- or double-layer suture closure of the rectus fascia using either absorbable or nonabsorbable suture material.^3,8,9^

There is currently a debate as to whether absorbable vs nonabsorbable rectus plication accomplishes the lowest rate of recurrence. Early studies investigating the use of different suture materials found that there was residual or recurrent diastasis at long-term follow-up in as high as 40% of patients who underwent plication with vicryl sutures.^10^ However, vicryl sutures have a short-lasting, well-known absorption profile with complete hydrolysis between 56 and 70 days, which is shorter than many other available absorbable suture materials.^11^ Polydioxanone suture (PDS) is an alternate longer lasting, absorbable suture material that does not fully absorb until around 120 days.^12^

The use of nonabsorbable vs absorbable suture material has been a topic of research in general surgery for many years with respect to its use for hernia repairs. An in-depth review of several prospective studies assessing outcomes when using nonabsorbable vs absorbable suture for hernia repairs without mesh found that there was no significant difference in outcomes between the 2 materials.^13^ Several other older publications have also supported these findings and have suggested that longer lasting absorbable sutures, such as PDS, did not degrade before the wounds had acquired sufficient strength for the repair.^14-16^ However, the question must be asked of whether or not these findings are applicable to rectus sheath plication performed during abdominoplasty.

Several recent studies have demonstrated success with the use of absorbable sutures for rectus sheath plication during abdominoplasty.^17^ A prospective study of 12 patients who underwent rectus plication with double-layer 0-polydioxanone suture found no recurrence of diastasis on CT imaging at long-term follow-up.^18^ In another prospective study of 51 patients undergoing plication with polydioxanone sutures, ultrasound imaging at an average follow-up of 20.8 months found that the use of absorbable sutures is a reliable method that maintains long-term stability.^8^

Although the success with the use of long-lasting absorbable sutures was reported in a more recent literature, many of these studies are limited by a combination of retrospective methodology, a small sample size, or short-term follow-up.^19^ This has led to continued debate about the true efficacy of absorbable sutures for rectus sheath plication. However, although there has been continued debate, it is important to acknowledge that there are benefits in using absorbable sutures for plication. The use of absorbable sutures eliminates the need for permanent suture material that may result in visible or palpable suture knots, suture pull-through, or material that may act as a focus for infection. Additionally, nonabsorbable sutures are more prone to the development of cysts and are more likely to cause postoperative pain.^20-22^

Although the use of absorbable sutures for rectus sheath plication does have identifiable benefits for the patient, the long-term durability of rectus diastasis correction with absorbable sutures must be further explored through continued research. The goal of this study is to review a single surgeon's experience with absorbable suture rectus plication.

## METHODS

### Patients and Data

This study received IRB approval from Northwestern University IRB (#STU00216721). A retrospective review was performed of all patients who underwent abdominoplasty with rectus plication, using absorbable sutures by the senior author from June 2018 to October 2022. Patients were excluded from analysis if they did not have at least a 6-month follow-up in order to assess diastasis recurrence. Demographic information, medical comorbidities, surgical history, operative details, including concurrent procedures, and postoperative complications were all obtained from the electronic medical record. Patient consent was obtained for the use of representative postoperative images.

### Surgical Technique

**Figure 1.**
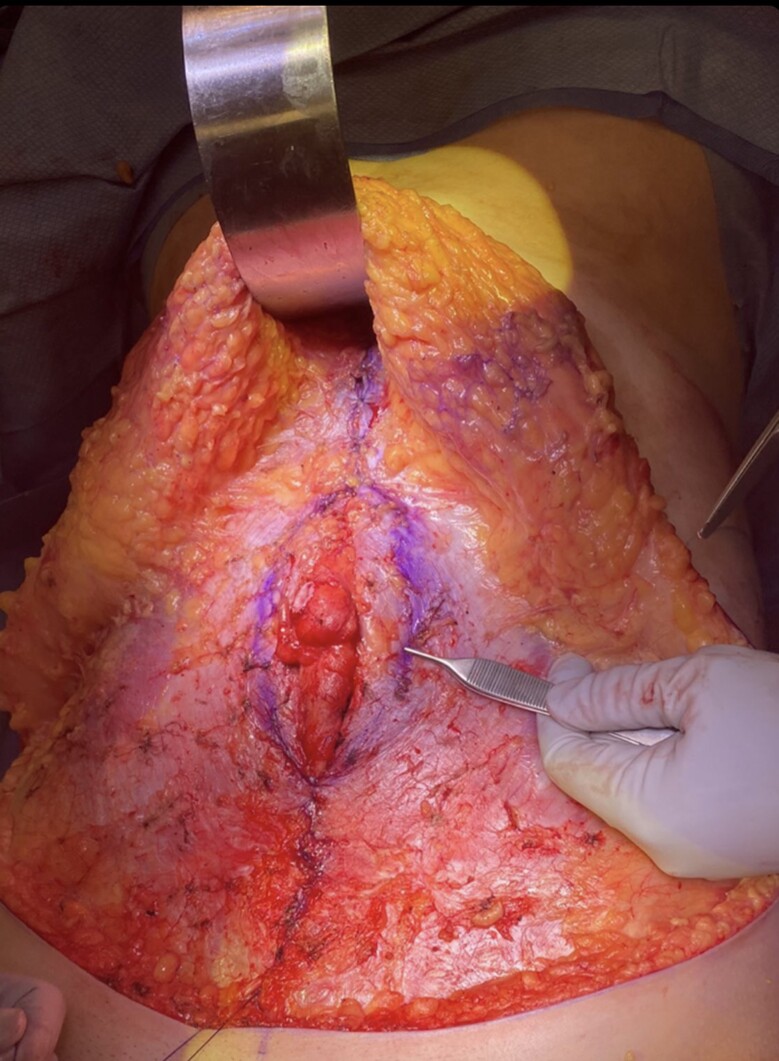
Markings for rectus plication with partial closure. The medial borders of the rectus muscles are identified, and the area is drawn in an ellipse.

**Figure 2.**
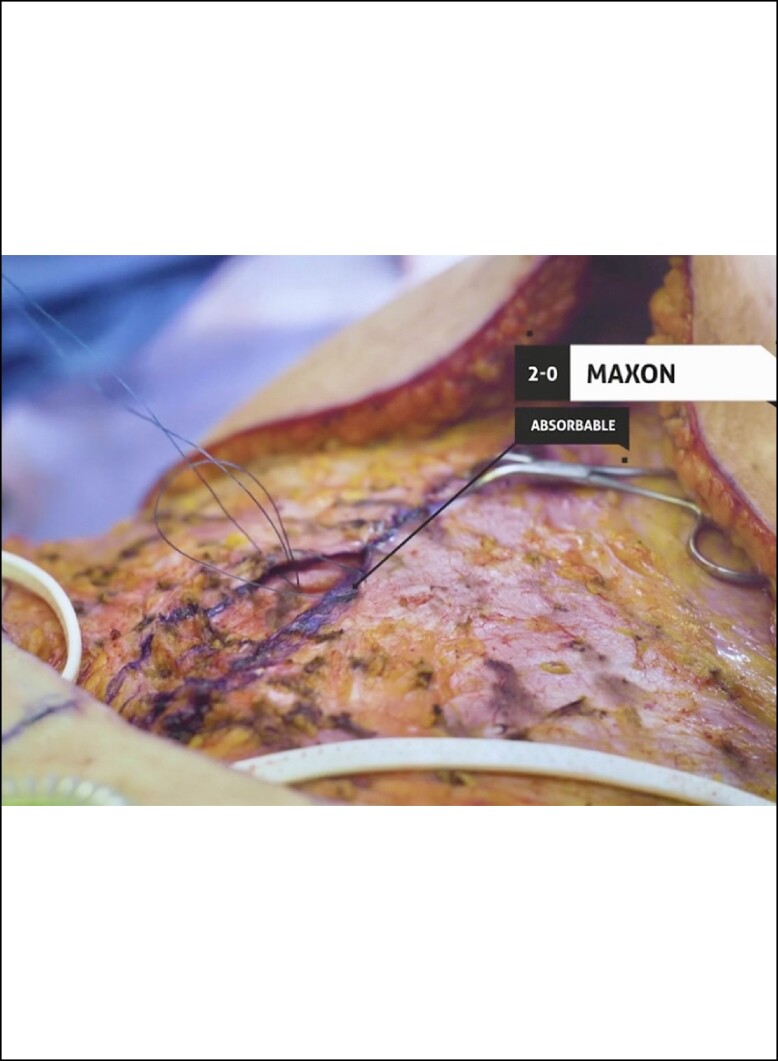
First layer of the interrupted figure of 8 sutures.

**Figure 3.**
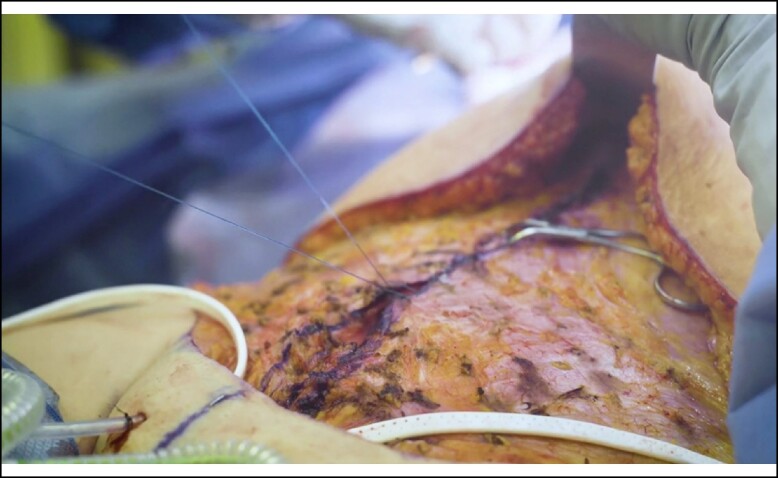
First layer of the interrupted figure of 8 sutures.

**Figure 4.**
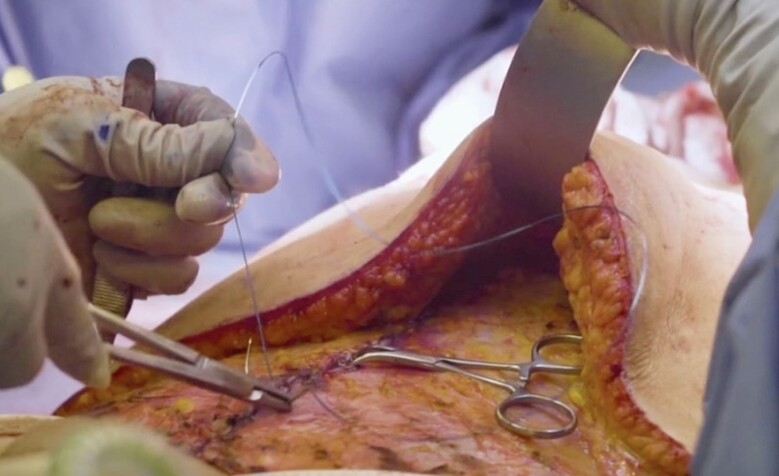
Second layer of sutures in running fashion using a #0 looped Maxon or PDS.

**Figure 5.**
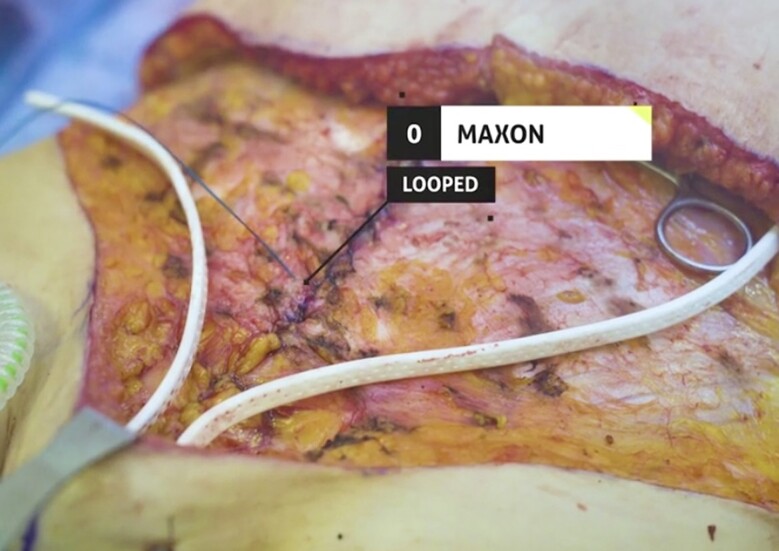
Second layer of sutures in running fashion using a #0 looped Maxon or PDS.

A full video demonstration of the senior author's technique is provided in Video. After elevation of the abdominal flap to the xiphoid centrally and just beyond the anterior costal margins, the entire central anterior sheath is exposed. The medial borders of the rectus muscles are marked with a surgical marking pen from xiphoid down to pubis forming an ellipse (Figure 1). If the medial borders are difficult to identify, bovie electrocautery can be used to see where muscle contraction stops medially. This delineates the lateral edge of the linea alba. After markings are drawn, interrupted, buried, figure of 8 stitches is placed ∼1 cm apart using 2-0 Maxon (Covidien, Mansfield, MA) or PDS (Ethicon, Somerville, NJ; Figures 2, 3). A second layer is then performed in running fashion using a #0 looped Maxon or PDS (Figures 4, 5).

### Assessment of Rectus Diastasis

Postoperative recurrence of rectus diastasis was assessed using physical examination. The patient was initially asked to lay flat on the examination table and then flex into a sustained sit up position at 30°. Manual palpation was then performed by the senior author to assess for diastasis and the degree of separation.

### Statistical Analysis

Data analysis was performed using IBM's SPSS (Armonk, NY). Descriptive statistics are displayed as means and ranges. Categorical data are displayed as numbers and percentages.

## RESULTS

Seventy-one female patients who underwent abdominoplasty with absorbable suture plication were included in this study. The average follow-up time was 21.1 months (range, 6-64 months). The average age at the time of surgery was 43.3 years (range, 27-65 years). Mean weight and BMI were 71.8 kg (range, 45.0-128.2 kg) and 26.8 (range, 17.5-40.6), respectively. Our patient population was a relatively healthy cohort, but relevant medical comorbidities included 4.2% with diabetes, 7% with hypertension, and 1.4% current smoker. Fifty-five patients (77.5%) had prior pregnancies, with 29 patients (41%) having prior Cesarean section and the average number of birthed children was 2 (range, 0-4). Approximately, two-thirds of the patients had prior abdominal surgery (*n* = 43, 61%). One patient had a prior abdominoplasty and presented for revision. The full demographic profile of our study cohort is summarized in Table 1.

**Table 1.** Patient Demographics

Average age	43.3 years (range, 27-65 years)
BMI	26.8 kg/m^2^ (range 17.5-40.6)
Sex	
Female	71 (100%)
Ethnicity	
African American	3 (4.2%)
Asian	3 (4.2%)
Caucasian	50 (70.4%)
Hispanic	12 (16.9%)
Not recorded	3 (4.2%)
Diabetes	3 (4.2%)
Hypertension	5 (7%)
Smoking	
Current	1 (1.4%)
Former	13 (18.3%)
Never	57 (80.3%)
Prior pregnancy	55 (77.5%)
C-section	29 (40.8%)
Average children birthed	2.0 (range, 0-4)
Prior abdominal surgery	43 (60.6%)
Average follow-up	21.1 months (range, 6-64 months)

In terms of intraoperative details, the average surgical duration was 248.5 min (ranges, 90-635 min). Twenty-two patients (31%) had a concurrent breast procedure performed at the time of abdominoplasty, including augmentation, mastopexy, reduction, implant exchange, nipple reconstruction, or a combination. All patients (100%) had rectus muscle plication with absorbable sutures in 2 layers, as described in the Methods section (Table 2).

**Table 2.** Operative Details

Average estimated blood loss	81.4 cc (range, 20-200 cc)
Surgical duration	248.5 min
Concurrent breast procedure	22 (31%)
Absorbable mesh overlay	1 (1.4%)

Complications included delayed wound healing (11%), seroma (8.5%), hematoma (2.8%), and pulmonary embolism (2.8%), which are presented in Table 3. No patients who had a rectus plication needed reoperation (0% revision rate). No patients had a recurrence of diastasis (0% diastasis recurrence rate) with up to 3 years follow-up.

**Table 3.** Complications

Complication	*n* (%)
Delayed wound healing	8 (11)
Seroma	6 (8.5)
Hematoma	2 (2.8)
Cellulitis	0 (0)
DVT/PE	2 (2.8)
Diastasis recurrence	0 (0)
Revision rate	0 (0)

DVT, deep vein thrombosis; PE, pulmonary embolus.

**Figure 6.**
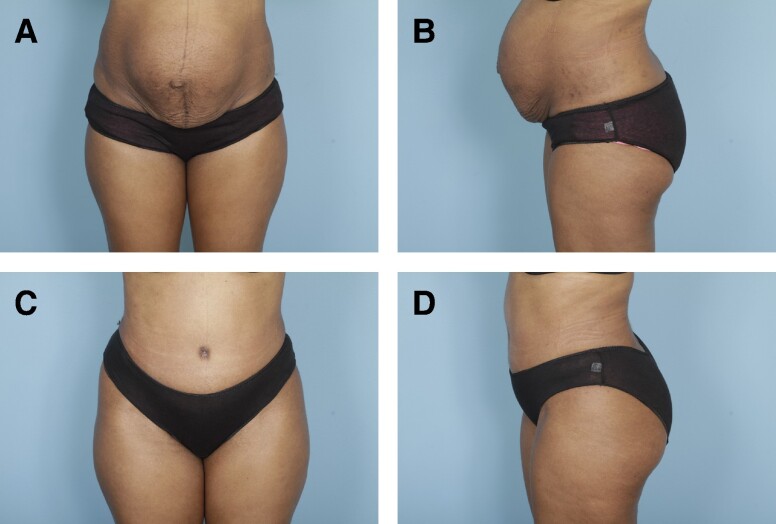
(A, B) Preoperative photographs of a 33-year-old female with a BMI of 26.0. (C, D) Six-month postabdominoplasty with rectus plication.

**Figure 7.**
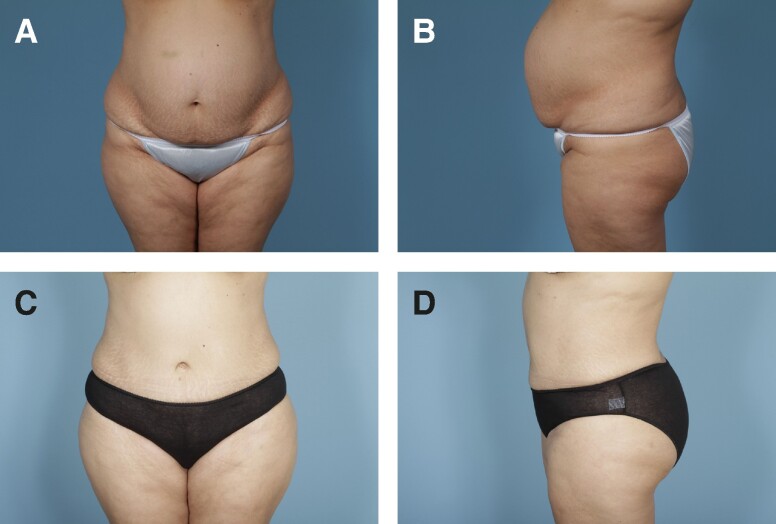
(A, B) Preoperative photographs of a 47-year-old female with a BMI of 27.0. (C, D) One-year postabdominoplasty with rectus plication.

**Figure 8.**
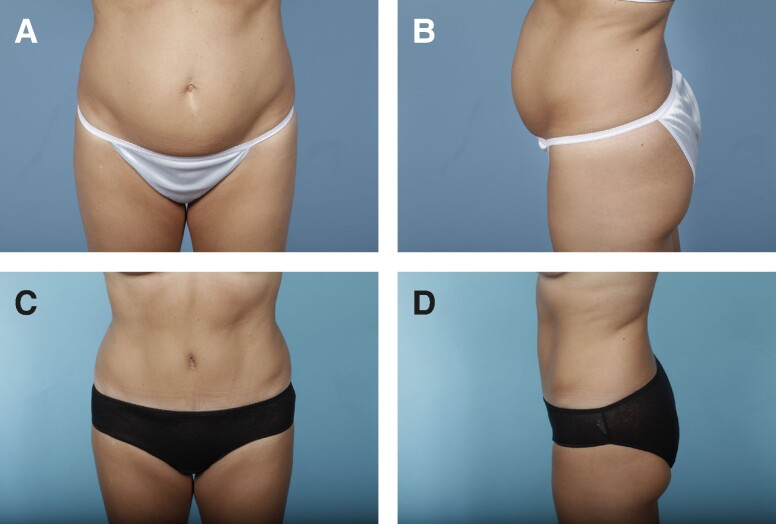
(A, B) Preoperative photographs of a 42-year-old female with a BMI of 24.9. (C, D) Six-month postabdominoplasty with rectus plication.

**Figure 9.**
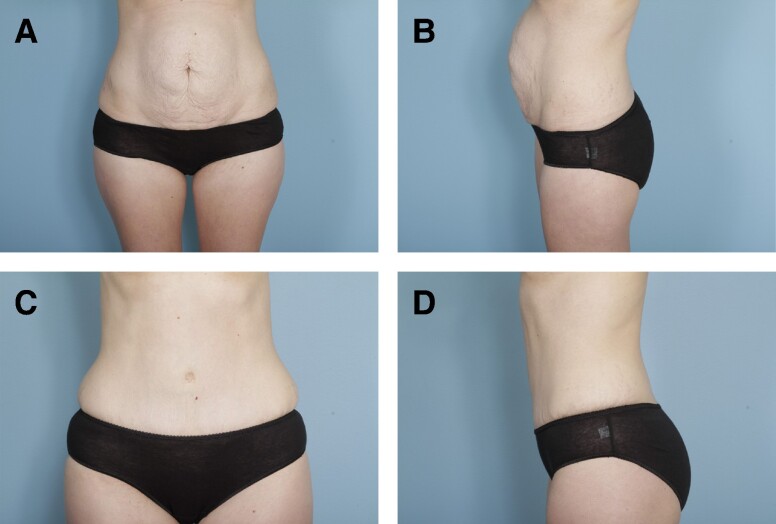
(A, B) Preoperative photographs of a 34-year-old female with a BMI of 19.7. (C, D) Two-year postabdominoplasty with rectus plication.

**Figure 10.**
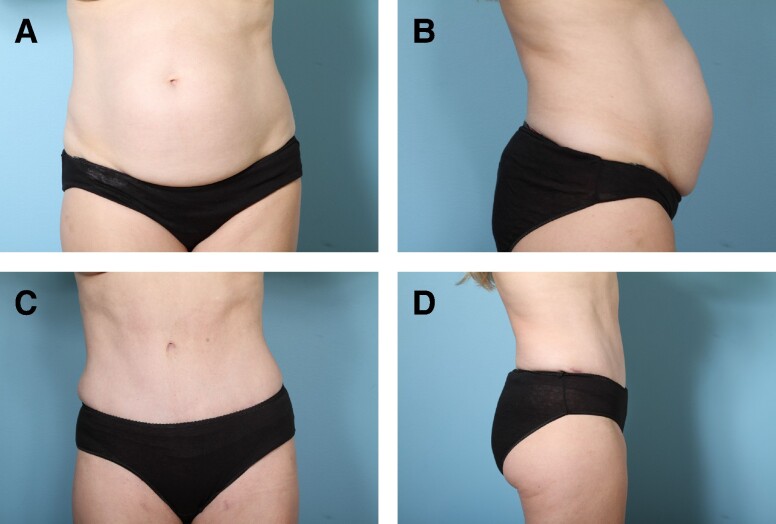
(A, B) Preoperative photographs of a 44-year-old female with a BMI of 24.7. (C, D) Six-month postabdominoplasty with rectus plication.

Preoperative and postoperative photographs of 3 patients from our cohort who underwent abdominoplasty with rectus plication using the senior author's surgical technique are shown in Figures 6-10.

## DISCUSSION

Rectus diastasis is a common condition encountered in our field stemming from the pathologic widening of the linea alba due to the midline separation of the rectus abdominis muscles. It is both a functional and cosmetic problem, and surgical plication of the rectus sheath during an abdominoplasty is the standard treatment for this condition. We found that surgical correction of rectus diastasis with the author's double-layered absorbable suture technique was successful in all of the patients included in this analysis, without any recurrences measured by physical examination at postoperative visits with up to 3 years of follow-up. Our findings suggest this long-lasting absorbable suture technique can be a successful method to achieve long-term correction of rectus diastasis.

Long-term correction of rectus diastasis using absorbable sutures has been supported by recent literature. In their recent prospective study of 12 patients undergoing rectus plication with a double-layer 0-polydioxanone suture, Nahas et al observed no recurrences of diastasis measured by CT imaging at follow-up times ranging from 32 to 48 months.^18^ In another case–control study, Mestak et al compared the interrecti distances at 3 different levels of the linea alba in nulliparous patients vs patients who underwent diastasis correction with polydioxanone sutures using ultrasound imaging and they found that there was no statistically significant difference in interrecti distances between the studied group undergoing the diastasis repair and the control group of nulliparous women at a mean follow-up time of 20.8 months.^8^ The results of our study corroborate these findings, as we observed no recurrences of rectus diastasis in all of our patients who underwent diastasis correction with the senior author's polydioxanone suture technique. Taken together, these observations bolster support for the argument that plication with the polydioxanone suture technique can achieve long-term correction of rectus diastasis.

Another point to consider is the benefit of using absorbable sutures for plication due to their lower complication profile when compared with nonabsorbable sutures. Chalya et al suggested a relationship between nonabsorbable sutures and chronic pain in midline abdominal incisions and emphasized the marked inflammatory reaction caused by these sutures. Furthermore, nonabsorbable sutures may generate highly undesirable palpable nodules after diastasis correction.^22^ Nahas et al studied the different types of sutures utilized in plication and for correction of diastasis during abdominoplasty and concluded that there is sufficient data to recommend slowly absorbable sutures of 0 polydioxanone for correction of abdominal diastasis. They also acknowledge that nonabsorbable sutures are more prone to develop granulomas and cause postoperative pain.^17^ In a retrospective analysis of 34 patients who underwent diastasis repair with either barbed or smooth absorbable polydioxanone sutures, Rosen and Hartman found no cases of recurrent diastasis, a 6% seroma rate, and a 3% hematoma rate after 34 months of follow-up.^20^ Similarly, we found a low complication profile with our absorbable suture technique for abdominoplasty plication.

In our study, we had 8 patients with delayed wound healing (11%) who was treated with local wound care. Six patients had seroma (8.5%) requiring aspiration in clinic and 2 patients had delayed hematoma (2.8%) that were drained in clinic. No patients required return to the operating room for any reason, and there were no recurrences of rectus diastasis observed with our absorbable suture plication technique. These lasting results with absorbable sutures are likely due to the fact that we are observing the impact of sufficiently strong tissue regeneration postsuture absorption.^23,24^ The suture material used in this investigation is fully absorbed by 120 days, so with our minimum follow-up of 6 months, we are observing the strength of this soft-tissue regeneration.^12^ Altogether, these findings suggest that the use of longer acting absorbable sutures for diastasis repair is not only safe but is also associated with a low complication profile.

The strengths of this study are that it is a large, consecutive, single surgeon study, with a relatively homogenous study population. This confers an acceptable degree of uniformity with respect to the absorbable suture technique used for diastasis correction, the cohort undergoing the repair, and postoperative assessment of successful correction. However, although single surgeon studies allow for conformity for technique and postoperative assessment, they do lack data from multiple clinical sites and may therefore limit the generalizability of this technique to other plastic surgeons. Other limitations of this study include the fact that unlike prior studies supporting the use of absorbable sutures for rectus diastasis repair, no radiographic analysis was used to evaluate the correction of the repair. Although the postoperative physical examination assessment by the senior author provides a consistent method of evaluation, the addition of radiographic imaging would have provided an additional, objective measurement of the success of the repair. It should be noted though that the physical examination assessment for diastasis recurrence was performed at postoperative visits, and this method of assessment does have improved fidelity due to the modification of subcutaneous tissue by liposuction and/or direct lipectomy during the abdominoplasty. Another limitation is that our study did not include any patient reported outcome measurements, which could have provided information regarding the study population's satisfaction with the repair. However, it is worth noting that despite the strong cosmetic improvement achieved with diastasis correction, few reports include validated instruments of satisfaction and patient outcome for this type of procedure. Third, despite relatively uniform demographic characteristics among the study population, it is reasonable to suggest that each patient's aponeurosis may be unique in terms of its collagen and metalloproteinase content.^21^ This contention is particularly relevant to our study population, as the durability of all absorbable suture repair techniques relies on the fibroplasia of each patient after the repair for long-term correction. Finally, it should be acknowledged that this investigation lacks a control group of patients who did not undergo plication with absorbable sutures. Although the outcomes of this study can be assessed in relation to historical data and prior investigations, there is no control cohort with which these patients can be directly compared.

In summary, our findings provide additional support that long-term correction of rectus diastasis can be achieved with long-lasting absorbable sutures. We found that the surgical repair with double-layered absorbable suture technique was successful in all of our patients, without any recurrences in patients with >6 months of follow-up. Future studies should include prospective analysis comparing patients undergoing diastasis repair with absorbable sutures vs patients undergoing repair with nonabsorbable sutures.

## CONCLUSIONS

Abdominal wall plication using a double-layered, long-lasting absorbable suture closure, utilized in the manner described in this paper, is a safe, reliable, and effective method to address rectus diastasis during abdominoplasty. Our technique achieved no clinical recurrence of diastasis in any patient and a low complication profile.

## References

[1] ElHawary H, Abdelhamid K, Meng F, Janis JE. A comprehensive, evidence-based literature review of the surgical treatment of rectus diastasis. Plast Reconstr Surg. 2020;146(5):1151–1164. doi: 10.1097/PRS.0000000000007252 33136963

[2] Rath AM, Attali P, Dumas JL, Goldlust D, Zhang J, Chevrel JP. The abdominal linea alba: an anatomo-radiologic and biomechanical study. Surg Radiol Anat. 1996;18(4):281–288. doi: 10.1007/BF01627606 8983107

[3] Nahabedian MY. Management strategies for diastasis recti. Semin Plast Surg. 2018;32(3):147–154. doi: 10.1055/s-0038-1661380 30046291 PMC6057788

[4] Brauman D. Diastasis recti: clinical anatomy. Plast Reconstr Surg. 2008;122(5):1564–1569. doi: 10.1097/PRS.0b013e3181882493 18971741

[5] de Almeida Mendes D, Nahas FX, Veiga DF, et al Ultrasonography for measuring rectus abdominis muscles diastasis. Acta Cir Bras. 2007;22(3):182–186. doi: 10.1590/s0102-86502007000300005 17546290

[6] Gama LJM, Barbosa MVJ, Czapkowski A, Ajzen S, Ferreira LM, Nahas FX. Single-layer plication for repair of diastasis recti: the most rapid and efficient technique. Aesthet Surg J. 2017;37(6):698–705. doi: 10.1093/asj/sjw263 28333252

[7] Jessen ML, Öberg S, Rosenberg J. Treatment options for abdominal rectus diastasis. Front Surg. 2019;6:65. doi: 10.3389/fsurg.2019.00065 31803753 PMC6877697

[8] Mestak O, Kullac R, Mestak J, Nosek A, Krajcova A, Sukop A. Evaluation of the long-term stability of sheath plication using absorbable sutures in 51 patients with diastasis of the recti muscles: an ultrasonographic study. Plast Reconstr Surg. 2012;130(5):714e–719e. doi: 10.1097/PRS.0b013e318267d806 23096625

[9] Yousif JN, Lifchez SD, Nguyen HH. Transverse rectus sheath plication in abdominoplasty. Plast Reconstr Surg. 2004;114(3):778–784. doi: 10.1097/01.prs.0000131023.09405.a8 15318062

[10] van Uchelen JH, Kon M, Werker PM. The long-term durability of plication of the anterior rectus sheath assessed by ultrasonography. Plast Reconstr Surg. 2001;107(6):1578–1584. doi: 10.1097/00006534-200105000-00046 11335840

[11] Akin Global Medical. Ethicon-coated VICRYL (Polyglactin 910) suture. Accessed January 23, 2024. https://www.akinglobal.com.tr/uploads/subdir-235-4/ETHICON-Coated-VICRYL-Suture.pdf.

[12] Lin HL, Chu CC, Grubb D. Hydrolytic degradation and morphologic study of poly-p-dioxanone. J Biomed Mater Res. 1993;27(2):153–166. doi: 10.1002/jbm.820270204 8436572

[13] Dobrin PB, et al Suture selection for hernia repair. In: Bendavid R Abrahamson J, Arregui ME, eds. Abdominal Wall Hernias. Springer; 2001:237–245.

[14] Dick AC, Deans GT, Irwin ST. A prospective study of adult inguinal hernia repairs using absorbable sutures. J R Coll Surg Edib. 1996;41(5):319–320. 8908956

[15] Nordin P, Haapaniemi S, Kald A, Nilsson E. Influence of suture material and surgical technique on risk of reoperation after non-mesh open hernia repair. Br J Surg. 2003;90(8):1004–1008. doi: 10.1002/bjs.4122 12905556

[16] Hilgert RE, Dorner A, Wittkugel O. Comparison of polydioxanone (PDS) and polypropylene (Prolene) for Shouldice repair of primary inguinal hernias: a prospective randomized trial. Eur J Surg. 1999;165(4):333–338. doi: 10.1080/110241599750006866 10365834

[17] Nahas FX, August SM, Ghelfond C. Nylon versus polydioxanone in the correction of rectus diastasis. Plast Reconstr Surg. 2001;107(3):700–706. doi: 10.1097/00006534-200103000-00008 11304594

[18] Nahas FX, Ferreira LM, Ely PB, Ghelfond C. Rectus diastasis corrected with absorbable suture: a long-term evaluation. Aesthet Plast Surg. 2011;35(1):43–48. doi: 10.1007/s00266-010-9554-2 21108036

[19] Tadiparthi S, Shokrollahi K, Doyle GS, Fahmy FS. Rectus sheath plication in abdominoplasty: assessment of its longevity and a review of the literature. J Plast Reconstr Aesthet Surg. 2012;65(3):328–332. doi: 10.1016/j.bjps.2011.09.024 22015165

[20] Rosen A, Hartman T. Repair of the midline fascial defect in abdominoplasty with long-acting barbed and smooth absorbable sutures. Aesthet Surg J. 2011;31(6):668–673. doi: 10.1177/1090820X11415242 21813880

[21] Nahas FX, Faustino LD, Ferreira LM. Abdominal wall plication and correction of deformities of the myoaponeurotic layer: focusing on materials and techniques used for synthesis. Aesthet Surg J. 2019;39(Supplement_2):S78–S84. doi: 10.1093/asj/sjy333 30869750

[22] Chalya PL, Massinde AN, Kihunrwa A, Mabula JB. Abdominal fascia closure following elective midline laparotomy: a surgical experience at a tertiary care hospital in Tanzania. BMC Res Notes. 2015;8(1):281. doi: 10.1186/s13104-015-1243-4 26121978 PMC4486392

[23] Li Y, Meng Q, Chen S, et al Advances, challenges, and prospects for surgical suture materials. Acta Biomater. 2023;168:78–112. doi: 10.1016/j.actbio.2023.07.041 37516417

[24] Ray JA, Doddi N, Regula D, Williams JA, Melveger A. Polydioxanone (PDS), a novel monofilament synthetic absorbable suture. Surg Gynecol Obstet. 1981;153(4):497–507. 6792722

